# Comparative study on the accuracy, safety and clinical effect of CT navigation and traditional open screw placement in the Treatment of Thoracic Fracture

**DOI:** 10.12669/pjms.39.4.3925

**Published:** 2023

**Authors:** Yang Song, Yue Ma, Fan Li

**Affiliations:** 1 Yang Song, Department of Orthopaedic, Affiliated Hospital of Beihua University, 132011, Jilin, P. R. China; 2 Yue Ma, Department of Orthopaedic, Affiliated Hospital of Beihua University, 132011, Jilin, P. R. China; 3 Fan Li, Department of Orthopaedic, Affiliated Hospital of Beihua University, 132011, Jilin, P. R. China

**Keywords:** Thoracic fracture, CT navigation, traditional open surgery, pedicle screw

## Abstract

**Objective:**

To explore the accuracy, safety and clinical effect of descending thoracic pedicle screw fixation assisted by computer CT three-dimensional navigation in the treatment of single segmental compression thoracic fracture.

**Methods:**

This study was a retrospective analysis. From June 2020 to June 2022, eighty patients with thoracic vertebral fractures admitted to Affiliated Hospital of Beihua University were were divided into observation group and control group according to different methods of screw placement, with 40 cases in each group. The navigation system was used to insert pedicle screws, and the control group used traditional open X-ray to insert pedicle screws by hand. Further comparison was carried out in terms of the operation time, intraoperative blood loss, perioperative complications, accuracy and safety rate of screw placement, and vertebral compression ratio between both the groups.

**Results:**

The average intraoperative blood loss in the observation group was significantly less than the control group, the average screw insertion time was significantly shorter than the control group, the postoperative average vertebral body compression ratios was significantly better than the control group, the excellent rate of screw insertion was better than the control group, while the incidence of complications was lower than the control group, and the difference was statistically significant (all P<0.05).

**Conclusion:**

Intraoperative CT navigation for pedicle screw placement can reduce the time of screw placement and intraoperative blood loss, improve the excellent rate of screw placement and the compression ratio of the anterior edge of the injured vertebra, the complication rate was low.

## INTRODUCTION

With the increase of construction accidents and traffic accidents, the incidence of thoracic fractures is on the rise, accounting for about 30.94% of spinal fractures, and has become a common type of spinal fracture.^1^,^2^ The concept of treating thoracic vertebral fractures is to restore the sequence and mechanical stability of the vertebral body. If conservative treatment is adopted, the recovery time of patients will be long and the result will be poor.

At present, surgery is the conventional method for treating thoracic vertebral fractures, and pedicle screw fixation is a commonly used internal fixation operation, can restore the spine alignment, strong biological stability.^3^ Compared with the lumbar spine, the pedicles of the thoracic spine are smaller, have more important tissues around them, are in close contact with the spinal dural sac, and have main blood vessels such as the thoracic aorta in front of the thoracic spine, nerves, blood vessels. Although the traditional open surgery has a clear surgical field of vision and simple nail placement, the damage is relatively large and the postoperative recovery of the patient is slow.^4^,^5^ With the development of technology and the introduction of CT navigation technology, real-time and clear three-dimensional images can be provided during nail placement, which improves the accuracy of nail placement.

At present, there are few clinical reports comparing CT navigation and traditional open pedicle screw placement in thoracic fracture pedicle screw placement. This study aimed to compare the effect of CT navigation and traditional open pedicle screw placement on single-segment thoracic compression Accuracy, safety and clinical efficacy of sexual fractures.

## METHODS

In this study 80 patients with single-segment thoracic vertebral compression fractures who were treated in Affiliated Hospital of Beihua University from June 2019 to June 2022. They were divided into CT navigation group (observation group) and traditional open group (control group) according to the operation method, 40 cases in each group.

### Ethical Approval:

This study was approved by the Institutional Ethics Committee of Affiliated Hospital of Beihua University (No.:2023[25]; May 18, 2023), and written informed consent was obtained from all participants.

### Inclusion criteria:

- Fresh single-segment thoracic vertebral compression fracture (fracture time <3 weeks), with or without neurological symptoms.
- Age 18-60 years old.
- Complete clinical data.

### Exclusion criteria:

- Multi-segmental fracture, Burst fracture.
- Accompanied by other bone-related diseases, such as scoliosis, bone tumors, and spinal tuberculosis.
- There are surgical contraindications.

Operations in both groups were performed by the same group of doctors in strict accordance with the operating standards. All patients were under general anesthesia in prone position.

### Observation group:

The reference frame was installed on the spinous process of the adjacent vertebral body that did not interfere with the operation process, the mirror was placed within the receiving range of the binocular infrared camera, CT scan was performed, and the image scanning results were input into the image navigation workstation. Use the three-dimensional navigation mode was used to puncture with a puncture needle with a hollow cannula under direct vision, determine the needle entry point and angle, pull out the inner core and insert the guide wire, make a one cm long incision, blunt dissection, and then use the step-by-step expansion channel system, choose the appropriate Pedicle screws which are placed and pre-bent rods are installed, and the nuts are tightened after the pedicle screws are stretched. During the whole process of pedicle screws insertion, the accuracy of the pedicle screws path was repeatedly verified by using the navigation inner core, and finally the position of the internal fixation was confirmed by fluoroscopy, and the pedicle screws insertion operation was completed. Control group: The injured vertebra and its upper and lower vertebral bodies were exposed after incision through the posterior median approach, and the upper and lower vertebral bodies were exposed layer by layer. The C-arm X-ray machine was positioned in the positive and lateral position of the thoracic spine, and the pedicle screws were inserted by hand, and the pre-bent rod was installed, after stretching the pedicle screws, pressurize and tighten the nuts. Determine the height recovery of the injured vertebra under fluoroscopy based on the height of the adjacent vertebra. After confirming satisfaction, the incision was flushed and sutured layer by layer, and the operation was completed. Both groups signed the operation informed consent. Besides, the operation of both groups was completed by the same team of doctors.

### Statistical indicators:

Perioperative conditions: The intraoperative blood loss, screw insertion time and postoperative complication rate of the two groups**.**

***Grade of screw placement:*** Mobbs-Raley classification^6^ was used to evaluate;

***Grade-0:*** The screw completely inserted into the pedicle cortex;

***Grade-1:*** The screw enters the pedicle wall less than 2 mm;

***Grade-2:*** The screw enters the pedicle wall more than 2 mm, but without nerve injury;

***Grade-3:*** The screw thread penetrates the pedicle wall, accompanied by complications such as pedicle fracture, screw penetration injury to anterior blood vessels and nerves, and sequelae of penetration injury to the medial or lateral nerves. Excellent rate=(grade-0+grade-1+grade-2)/total number of screws×100%.

Judging by the compression ratio of the vertebral body height, the vertebral height compression ratio = (average height of the anterior edge of adjacent vertebral bodies of the injured vertebra - height of the anterior edge of the injured vertebra)/average height of the anterior edge of adjacent vertebral bodies of the injured vertebra×100%. The postoperative compression ratio of the injured vertebrae was reviewed three months after operation.

### Statistical analysis:

SPSS23.0 software was used for data entry and statistical analysis. The measurement data were represented by mean ± standard deviation (χ±s), and the comparison between groups is by independent sample t-test. The count data were represented by the percentage of cases [n(%)], and the comparison between groups is by Chi-Square test. The inspection level was preset as *P*-value of 0.05.

## RESULTS

The observation group had 40 patients, including 19 males and 21 females, with an average age of 51.58±8.39 years. Meanwhile, there were 40 patients in the control group, including 20 males and 20 females, with an average age of 51.18±8.39 years old. there was no significant difference in the general data of the two groups of patients (P>0.05), which were comparable. Table-I.

**Table-I:** Baseline data of two groups of patients.

	Observation group(n=40)	Control group(n=40)	t/c^2^	P
Age(years)	51.58±8.39	51.18±7.22	0.229	0.820
Gender(male/female)	19/21	20/20	0.050	0.823
Causes of fractures			0.528	0.913
Falling injury	14(35.00)	13(32.50)		
Car accident	12(30.00)	15(37.50)		
Heavy object	8(20.00)	7(17.50)		
Others	6(15.00)	5(12.50)		

The average screw insertion time in the observation group was 8.43±2.63min; the average intraoperative blood loss was 92.65±8.10mL; a total of 192 screws were inserted, with an excellent rate of 96.35%; the average preoperative and postoperative vertebral body compression ratios were 50.90±7.91% and 90.24±1.79%. The average screw insertion time in the control group was 11.28±3.74min; the average intraoperative blood loss was 122.03±7.41mL; a total of 208 screws were inserted, with an excellent rate of 90.87%; the average vertebral body compression ratios before and after operation were 52.80±7.79% and 83.51±2.42%. There was no significant difference in the compression ratio of the anterior edge height of the injured vertebra between the two groups before operation (t=1.081, P>0.05). The average intraoperative blood loss in the observation group was significantly less than that in the control group, and the average screw insertion time was significantly shorter than that in the control group. The excellent rate of screw placement was higher than that of the control group, while the incidence of complications was lower than that of the control group. The difference was statistically significant (all P<0.05). The results are shown in Table-II and III.

**Table-II:** Comparison of perioperative indicators, vertebral height compression ratio, and complications between two groups.

	Observation group(n=40)	Control group(n=40)	t/χ^2^	P
Intraoperative blood loss (mL)	92.65±8.10	122.03±7.41	16.920	0.000
screw insertion time(min)	8.43±2.63	11.28±3.74	3.941	0.000
***complication [n (%)]***			4.021	0.045
Infect	1(2.50)	2(5.00)		
Incision non healing	0(0.00)	2(5.00)		
Crew loosening	2(5.00)	4(10.00)		
Venous thrombosis of lower limb	1(2.50)	3(7.50)		
***Vertebral height compression ratio (%)***				
Preoperative	50.90±7.91	52.80±7.79	1.081	0.283
Postoperative	90.24±1.79	83.51±2.42	14.153	0.000

**Table-III:** Comparison of nail placement between two groups [n (%)]

Group	Number screws inserted	0 Grade	1 Grade	2Grade	3 Grade	Excellent rate
Observation group(n=40)	192	159(82.81)	17(8.85)	9(4.69)	7(3.65)	185(96.35)
Control group(n=40)	208	143(68.75)	25(12.02)	17(8.17)	19(9.13)	189(90.87)
c^2^						4.949
P						0.026

## DISCUSSION

Due to the presence of the thorax, the stability of the thoracic spine is higher than that of the thoracolumbar and lumbar vertebrae.^7^ The fractures are mostly caused by high-energy violence, usually accompanied by organ damage and fractures in other parts, which may easily lead to misdiagnosis.^8^ Effective treatment can lead to various complications, which can lead to aggravation of the condition, which can threaten the life safety of the patient, is not conducive to the recovery of the patient, and increases the length of hospital stay and cost.^9^ Spinal fractures are mostly compression fractures, young people have mostly violent injuries, and patients with osteoporosis can occur without obvious incentives.^10^ After the fracture, the three-column structure of the vertebral body will be destroyed, affecting the stability of the spine. *Mild compression fractures:* The patient can be treated conservatively. Although damage to normal tissues can be avoided, the patient needs to wear a brace for a long time, which seriously affects the normal life of the patient, and the long-term effect is poor.^11^,^12^ Therefore, surgical treatment is required for most patients with thoracic vertebral fractures. Surgery can correct the collapsed and deformed parts of the vertebral body, restore its physiological structure, and maintain spinal stability and balance.^13^ At the same time, for patients with nerve damage, surgery can relieve nerve compression, which is conducive to the recovery of damaged nerve function and can effectively improve the prognosis of patients.^14^,^15^

The pedicles of the thoracic spine are small and the surrounding anatomical structure is complex. Inserting pedicle screws is more difficult than that of the lumbar spine.^16^,^17^ If the inner and outer walls of the pedicle, especially the inner wall, are broken through during screw insertion, the risk of nerve injury will increase. Although the traditional open screw placement surgery can improve the accuracy of screw placement, it destroys the surrounding normal tissues, resulting in long recovery time for patients and increasing the occurrence of complications.^18^ The C-arm is usually used in spinal surgery, but for obese and deformed patients, its effect will be affected, and frequent fluoroscopy is required during the operation, which increases the risk of incision infection and radiation exposure.^19^ CT navigation image quality is high, and the accuracy of nail placement is also high.^20^ In this study, the excellent and good rate of nail placement in the observation group was significantly higher than that in the control group (P<0.05); CT navigation can scan and evaluate in real time, which improves the safety of surgery and speeds up the operation. The operation time was shortened, and the intraoperative bleeding was reduced. In this study, the intraoperative blood loss, the time of screw placement, and the compression ratio of the anterior edge of the injured vertebra after operation were all better than those of the control group (all P<0.05), which is consistent with the results of many clinical studies unanimous. However, CT navigation also has the following disadvantages: (1) The equipment is expensive and difficult to popularize; (2) If a fault occurs during the operation, it cannot continue to be used; (3) The operation is cumbersome, and precise matching is required before navigation.

### Limitations of the study:

Due to relatively small sample size, the test results may be affected to a certain extent. In future studiers, a prospective randomized controlled study can be further carried out to confirm our observations.

## CONCLUSION

Compared with traditional open surgery, the use of CT navigation can increase the accuracy of screw placement and improve the excellent rate of screw placement compared with the control group, the observation group has less intraoperative bleeding and shorter screw placement time. The incidence of postoperative complications is low, and the surgical incision range is small when using CT navigation, which reduces the damage to the posterior ligament complex of the spine and better maintains the integrity and stability of the spinal structure. High compression is better than recovery.

### Authors’ Contributions:

**YS** designed this study, prepared this manuscript, and are responsible, accountable for the accuracy and integrity of the work. **YM** collected and analyzed clinical data. **FL** Data analysis, significantly revised this manuscript.
